# Participatory epidemiology: the contribution of participatory research to epidemiology

**DOI:** 10.1186/s12982-017-0056-4

**Published:** 2017-02-10

**Authors:** Mario Bach, Susanne Jordan, Susanne Hartung, Claudia Santos-Hövener, Michael T. Wright

**Affiliations:** 1 Robert Koch Institute, Berlin, Germany; 2 Catholic University of Applied Sciences Berlin, Berlin, Germany

**Keywords:** Participatory epidemiology, Methodology, Participatory research, Health promotion, Capacity building, Context, Conceptual framework

## Abstract

**Background:**

Epidemiology has contributed in many ways to identifying various risk factors for disease and to promoting population health. However, there is a continuing debate about the ability of epidemiology not only to describe, but also to provide results which can be better translated into public health practice. It has been proposed that participatory research approaches be applied to epidemiology as a way to bridge this gap between description and action. A systematic account of what constitutes participatory epidemiology practice has, however, been lacking.

**Methods:**

A scoping review was carried out focused on the question of what constitutes participatory approaches to epidemiology for the purpose of demonstrating their potential for advancing epidemiologic research. Relevant databases were searched, including both the published and non-published (grey) literature. The 102 identified sources were analyzed in terms of comparing common epidemiologic approaches to participatory counterparts regarding central aspects of the research process. Exemplary studies applying participatory approaches were examined more closely.

**Results:**

A highly diverse, interdisciplinary body of literature was synthesized, resulting in a framework comprised of seven aspects of the research process: research goal, research question, population, context, data synthesis, research management, and dissemination of findings. The framework specifies how participatory approaches not only differ from, but also how they can enhance common approaches in epidemiology. Finally, recommendations for the further development of participatory approaches are given. These include: enhancing data collection, data analysis, and data validation; advancing capacity building for research at the local level; and developing data synthesis.

**Conclusion:**

The proposed framework provides a basis for systematically developing the emergent science of participatory epidemiology.

## Background

Epidemiology provides the empirical foundation for public health. In its history, the discipline has contributed in many ways to identifying various risk factors for disease and to promoting population health. Over the last decades, epidemiology has developed models utilizing genetic, population, and environmental data to create knowledge relevant for policy makers and public health practice [1–3]. This includes describing the characteristics of populations (e.g. community diagnosis) and individuals; identifying risks for disease as well as the factors associated with good health; and evaluating treatment approaches and public health programs. Epidemiology has refined its methods over time in order to provide more accurate measures to serve these various purposes [1].

However, some aspects of epidemiology are still in a process of development. The call voiced by Stallones [4] in 1980 for an expanded view on causation of disease is of continued concern [5–9]. Stallones’ critique can be summarized under two headings. First, a sometimes simplified view on the various antecedents of health can hinder complex modeling, what scholars have labeled *biomedical individualism* or *individualization of risk* [10, 11]. Second, the application of theories about the connection between risk exposure and outcome often remains incomplete (the so-called *black box paradigm*). As a consequence, there are difficulties within epidemiology in terms of integrating individual-level with social and environmental-level risk factors [12]. There are also problems identifying factors which are the result of interactions between individuals (e.g. community-based initiatives) and social or political organizations (e.g. public health programs) [13]. Finally, although the discipline takes into account several macrosocial determinants of health (e.g. urbanization, migration, corporate practices within industries) [14–16] and the social determinants of health [17], it still faces problems in explaining “the complexity of disease occurrence at various and interacting levels” [18, p. 218]. Consequently, these challenging issues can cause problems in knowledge translation and public health program development [19–21] and thus raise the question whether epidemiology is still the basic science of public health [22]. It has been asked what would be necessary to preserve and advance the role of epidemiology [23].

We suggest participatory research as a way to enrich epidemiologic methodology in order to address some of these challenges. Participatory research has its “basis in broad social movements striving for a more democratic and inclusive society. There is a shared recognition that science is more than adherence to specific epistemological or methodological criteria; it is primarily a means for generating knowledge to improve people’s lives” [24, p. 5]. Over the past 20 years, several authors have called for more participation in epidemiologic research [6, 19, 25, 26]. Participatory forms of epidemiology are being conducted under various labels, including popular epidemiology [27], lay epidemiology [28], community-based research [29], and participatory health research [30]. The hallmark of participatory approaches in epidemiology are equitable research partnerships with a diverse group of stakeholders such as policy makers, public health professionals, health activists and community representatives from the populations “under study”. These partnerships are useful in identifying the causes of health problems and in finding strategies to address them.

The purpose of this paper is to illustrate the potential of participatory epidemiology for enhancing the quality and effectiveness of epidemiologic research through such partnerships. A scoping review was carried out on the question of how participatory approaches are being applied in epidemiology currently, contrasting these approaches with more common forms of epidemiologic research in the various stages of the research process. The result is a conceptual framework which researchers can use to advance study design and to inform their research practice. The framework provides principles to better address social complexity (e.g. the social and cultural determinants of health), contextual factors (e.g. environmental justice), as well to ways for disseminating findings so as to have a greater impact.

## Methods

To explore the emerging field of participatory approaches in epidemiology, a scoping review [31, 32] was conducted encompassing original studies, conceptual articles, book chapters, and reports (grey literature).

Firstly, a hand search conducted by the authors generated 71 manuscripts addressing participation in epidemiologic research. All manuscripts were read and relevant terms for a database search were extracted. This resulted in the following seven individual search terms: *participatory*, epidemiol*, *local*, *health*, *community*, health report*, indicator* and nine compound terms: *hard to reach*, *health promotion*, *health reporting*, *needs assessment*, *minority health*, *vulnerable groups*, *vulnerable population*, *peer research*, *and program evaluation*.

Secondly, six databases (EMBASE, SciSearch, PubMed, Scopus, Web of Science, Bielefeld Academic Search Engine) were searched (title, abstract, keywords) using different combinations of the search terms (AND operator), going back to 1970. After duplications were removed, 820 records remained. All titles and abstracts were screened using the following inclusion criteria: (1) the level or phase of participation is specified, (2) social and/or environmental determinants of health were taken into account, and (3) the relevance for public health practice was considered. This resulted in 102 sources being included in the development of our framework.

Thirdly, the included sources were analyzed based on the principles of a *realist synthesis*. Originally, realist synthesis was developed as an interpretative approach for studying interventions requiring a more complex assessment [33–35]. In our study, the approach was iteratively applied to appraise the evidence as found in the included sources with regard to *how* (e.g. collaboration with key stakeholders), *why* (e.g. goals like capacity building), and *where* (e.g. contextual factors) participatory approaches to epidemiology have been applied. More specifically, we investigated how the reported participatory approaches informed the design of the included studies with regard to the steps of the research process. Hence, we synthesized the identified participatory procedures to draw generalized conclusions on the approach which we subsumed under the label *participatory epidemiologic practice*.

Finally, a comparison between common research approaches and participatory research approaches in epidemiology were discussed and summarized (Table 2).

### Epidemiology and participation

We begin by providing an overview of the cited studies underpinning our framework with regard to the following aspects of the research process: defining the research goal, defining the research question, defining the population, reconsidering context, synthesizing heterogeneous data, managing the research process, and disseminating findings (Table 1). We then discuss each aspect by comparing common approaches to epidemiologic research to participatory approaches. At the end of this section, a summary of the framework with a point-by-point comparison is provided (Table 2).

**Table 1:** Selected original, conceptual, and methodological works

Section	Study title	Topic, context, and objective of research	Citation
Defining the research goal	Physicians, engineers, and urban health. Theories of the hygiene movement in the nineteenth century [transl. MB]	– Early engineering in sanitation of urbanized areas (Germany) – Informing interdisciplinary planning	Hardy [36]
	Why is an Integrated Social-Ecological Systems (ISES) lens needed to explain causes and determinants of disease? A case study of Dengue in Dhaka, Bangladesh	– Research on and prevention of dengue (municipality of Dhaka, Bangladesh) – Demonstrating the relevancy of multi-level modelling	Chowdhury and Emdad Haque [18]
	Engaging young adolescents in social action through photovoice: The Youth Empowerment Strategies (YES!) Project	– Report of an afterschool program on various health topics in an underserved population (schools and neighborhoods, California, USA) – Documenting social environments to tackle social action	Wilson et al. [37]
	Photovoice: concept, methodology, and use for participatory needs assessment	– Unspecific needs assessment in vulnerable population (international examples) – Reporting the pros and cons of photovoice for data collection	Wang and Burris [38]
Defining the research question	Participatory epidemiology: Use of mobile phones for community-based health reporting	– Local outbreak assessments with mobile applications (e.g. H1N1 virus) (on local levels, USA) – Complementing existing monitoring systems by crowdsourcing	Freifeld et al. [30]
	Improving sampling and response rates in children’s health research through participatory methods	– Research on the health status of students in a school setting (municipality of New York City, USA) – Demonstrating a community-centered approach	Claudio and Stingone [39]
	From asthma to AirBeat: community-driven monitoring of fine particles and black carbon in Roxbury, MA, USA	– Definition of local risk factors regarding asthma and air pollution (municipality of Roxbury, USA) – Integrating monitoring, communication and community education	Loh et al. [40]
	Development of a consumer constructed scale to evaluate mental health service provision	– Co-research on (face) validity and reliability of measures in mental health (university setting, Australia) – Improving mental health outcomes and satisfaction measures	Oades et al. [41]
	Local perceptions of cholera and anticipated vaccine acceptance in Katanga province, Democratic Republic of Congo	– Research on cholera prevention (province of Katanga, DR of Congo) – Investigating local and cultural attitudes and practices to enhance vaccination acceptance	Merten et al. [42]
	Thinking outside the box: Aboriginal people’s suggestions for conducting health studies with Aboriginal communities	– Culturally safe epidemiology with aboriginal communities (Province of Ontario, Canada) – Developing adequate research instruments and recruitment strategies	Maar et al. [43]
	Context and environment: the value of considering lay epidemiology	– Definition of risk for HCV infection among drug users (urban setting, Australia) – Accessing and synthesizing lay and expert knowledge	Olson and Banwell [28]
	Community-based participatory research in the California Health Interview Survey	– State level interview survey on population health (California, USA) – Integrating local and population-based data	Brown et al. [44]
Reconsidering context	The social and cultural context of risk and prevention: food and physical activity in an urban aboriginal community	– Cultural sensitive research on diabetes prevention (NIDDM) with aboriginal communities (municipality of Melbourne, Australia) – Developing and implementing acceptable prevention strategies	Thompson et al. [45]
	A participatory evaluation model for healthier communities: developing indicators for New Mexico	– Context oriented indicator development (State of New Mexico, USA) – Introducing a participatory mindset into modelling	Wallerstein [46]
	Culturally safe epidemiology: oxymoron or scientific imperative	– Co-defining the framework “culturally safe epidemiology” with First Nation communities, giving recommendations for usage (community-level, Canada)	Cameron et al. [47]
	Citizen deliberation in setting health-care priorities	– Review on deliberation practices in health care (United Kingdom) – Acknowledging individual values/criteria for prioritizing in health care	Murphy [48]
	Determinants of HIV, viral hepatitis and STI prevention needs among African migrants in Germany; a cross-sectional survey on knowledge, attitudes, behaviors and practices	– Assessment of knowledge, attitudes, behaviors, and practices in HIV, viral hepatitis, and STI with immigrants from sub-Saharan Africa (cities in Germany) – Creating an epidemiologic base of evidence for research and planning, community building and empowerment	Santos-Hövener et al. [49]
Synthesizing heterogeneous data	Community mapping and respondent-driven sampling of gay and bisexual men’s communities in Vancouver, Canada	– Assessment of gay/bisexual men networks (municipality of Vancouver, Canada) – Collecting data on social and sexual network characteristics	Forrest et al. [47, 50]
	Neighborhood mapping and evaluation: a methodology for participatory community health initiatives	– Evaluation of an urban infant mortality prevention program (municipality of Baltimore, USA) – Applying various data sorts to fully examine the influence of local contexts on health outcomes	Aronson et al. [51]
	The impact of regional and neighbourhood deprivation on physical health in Germany: A multilevel study	– Relationship between lower SES, unfavorable neighborhood conditions and individual health status (Germany) – Connecting area/neighborhood data with data on physical health	Voigtländer et al. [52]
	Enabling methods for community health mapping in developing countries	– Complementary population data (municipality of Bo, Sierra Leone) – Developing a geographic information system	Ansumana et al. [53]
Managing the research process	Community engagement in epidemiological research	– Description of community engagement activities in the National Children’s Study (USA) with regard to service development – Integrating community engagement into various steps of the research process	Sapienza et al. [26]
	Community-based epidemiology: Community involvement in defining social risk	– Co-developing the “risk concept” to inform research practice (neighborhood level, USA)	Smith [54]
	Development and implementation of a culturally sensitive cervical health survey: a community-based participatory approach	– Co-research on screening barriers in cervical health of Native Americans (Great Plains, USA) – Improving monitoring practices through a culturally sensitive approach	Smith et al. [55]
Disseminating findings	After epidemiological research: What next? Community action for health promotion	– Discussion of case studies on environmental health, HIV risk reduction, community betterment (neighborhood, Chicago) and other topics – Developing multidisciplinary networks and multimodal venues for communication and education	Goldsmith Cwikel [55, 56]
	Emerging communication responsibilities of epidemiologists	– Comprehensive recommendations for developing communicative spaces for epidemiologists and public health practitioners	Sandman [57]
	Dissemination as dialogue: Building trust and sharing research findings through community engagement	– Exploring social network characteristics for health outcomes among Black men and women living with HIV (municipality of Los Angeles, USA) – Creating a dialogical and adaptable knowledge dissemination strategy	McDavitt et al. [58]
	Community dissemination and genetic research: moving beyond results reporting	– Defining quality criteria for knowledge dissemination in ethnic groups (Alaska and Seattle, USA)	Trinidad et al. [2]

### Defining the research goal

Epidemiology has developed alongside changing concepts of health and disease, pursuing different goals for disease control and prevention, particularly for populations at risk [21, 59]. Historically, the definition of research goals in epidemiology has often referred to the wider “circumstances […] under which human disease is prone to develop” [60, p. 539]. But over time the definition narrowed, influenced by biomedical, causal, and downstream perspectives.

In the nineteenth century, a miasmatic paradigm of illness and death was predominant, accompanied by an epidemiology focused on the improvement of sanitation and the drainage of urban areas, often in collaboration with non-medical professionals, like engineers and local activists. Such endeavors are early examples for stakeholder participation that lay ground for collectively defining the goals of research, as is the case in a study on sanitation in urbanized areas in nineteenth-century Germany [36]. Later, the discovery of microbes led to a switch towards the germ paradigm which “opened the era of infectious disease epidemiology, in which epidemiologists have typically sought to relate a single agent to a specific disease” [61, p. 18]. Hence, the environmental and societal aspects of health were no longer the primary subject of epidemiologic research. With the rise of infectious disease epidemiology, the focus on pathogens, disease process, and the control of risk factors at the individual level has prevailed, constituting much of molecular, cell, and chronic disease epidemiology, as well [8, 20, 21]. This has been dominated by a “downstream” biomedical perspective on health problems, versus looking “upstream” to the contextual or social antecedents of health and disease [62]. More recently, there is renewed interest in the social determinants of health and in ecological modeling, with efforts to integrate upstream and downstream reasoning in study designs [18, 63].

Participatory approaches to epidemiology can contribute to defining research goals which are relevant for both academic researchers and public health practitioners, as they often focus on the antecedents of health and thus provide an empirical basis for achieving health equity through social and political action [64]. A good example is the Youth Empowerment Strategies (YES!) Anti-Violence Program in Richmond, California (USA). Young adolescences are empowered to take action on health topics which they identify using photovoice. Photovoice is a method by which people can identify various health-related issues through photographic techniques as a starting point for further research or community action. In a group-level research process, data is generated which is suitable for both qualitative and quantitative forms of analysis [38]. In YES! the adolescents produce data on the relationship between their environment and several dimensions of health-related behavior on which public health interventions can be based [65].

In summary, the common understanding of the research goal in participatory research lends itself to social and political outcomes. Thus, epidemiologists can profit by widening the scope and the aims of their studies.

### Defining the research question

Epidemiologic studies are usually conducted within academic institutions or by public health agencies in charge of safeguarding population health, the latter with the goal of providing evidence for health-related policy making. Hence, the definition of research questions and research priorities are typically driven by an academic agenda, which has been criticized as being too narrow [11]. There are several fields in epidemiology in which it is not immediately apparent how the research questions can be developed together with practitioners and the researched populations, for example, in clinical epidemiology. But even fields like genetics could profit from at least having their questions validated by practitioners, as shown in a project on the application of human genomic information for public health practice [66].

By applying a participatory approach, epidemiologists are able to identify the research questions that are meaningful to those immediately affected by the issue, providing knowledge which is more relevant for program development in public health [64, 67, 68]. In research partnerships, the generation of evidence is directly connected with advocacy and activism [30, 39]. People are empowered to have an influence on study design including the development of research questions [69]. For example, a participatory research design was applied in the community-driven monitoring study AirBeat on the links between high asthma rates and air pollution in a Boston (USA) neighborhood. Reliable data were produced on the long-term effects of air pollution at the community level [40]. The subsequent steps in the monitoring and communication process were planned in frequent meetings of researchers and community members. The AirBeat project illustrates how the identification of meaningful research questions in partnership with local people can itself be an integral part of the study design. Participatory research offers various methods to facilitate such processes [70].

However, there can be a tension between the desire of community groups to address broader issues and the focus of epidemiologists on highly specific issues of causality [71]. One possible solution is a sequencing of methods, as was shown in the participatory development of consumer constructed scales in the field of mental health. First, an interdisciplinary team of university-based researchers developed a first draft of scales. Second, consumers were involved in generating and validating the specific items. This is a quite common approach in consumer research, more generally, with consumers and researchers working as equal partners [41].

In summary, the definition of the research questions in participatory research is based on a consensus regarding the common goals of all project partners. This consensus rests on both the interests and needs of those involved and on the relevant ethical and political issues identified early in the research process [72, 73]. For epidemiologists, collaboratively defining the research question strongly challenges their own “role and legitimate boundaries” [74, p. 589] at the same time new opportunities for developing research studies arise.

### Defining the population

Defining the population under study is complex. Individual, social, cultural, and environmental factors represent only a small selection of possible characteristics, the interactions between them adding a further dimension. Commonly, a “case-centred epidemiology identifies individual susceptibility, but it may fail to identify the underlying causes of incidence” [75, p. 432]. Nevertheless, in many epidemiologic study designs, the researched populations are aggregated by individual attributes with the goal of answering questions regarding disease causation and distribution (*methodological individualism*) [76]. Recent developments in epidemiology seek to address this limitation. For example, multilevel modeling can include individual, societal (e.g. neighborhood) and regional characteristics [52]. Participatory epidemiologic research routinely makes use of characteristics that go beyond the individual level and can thus expand on other forms of modelling. For example, through a collaboration between epidemiology and anthropology, which has enhanced the recognition of local and tribal perceptions of health and disease as well as the recognition of factors triggered by globalization and emigration, thus providing sophisticated criteria to define the population under study [77, 78]. This practice avoids colonialist views on health and disease and thus can enhance the impact of public health interventions. Such an approach was recently reported in the field of vaccine acceptance in the Democratic Republic of Congo where local perceptions on health and culturally-based practices influenced the definition of the population under study [42]. Another example for participatory research in epidemiology is a culturally sensitive recruitment strategy that was developed with aboriginal communities in Ontario, Canada in the field of diabetes prevention [43].

The strengths of such approaches is the acknowledgement that “people live in complex, interconnected, and dynamic contexts” [79, p. 822]. Epidemiologists could benefit from recognizing this perspective when defining and seeking access to certain populations. This can help, for example, to improve the quality of the data on the perception and reporting of risks in specific communities, e.g. drug users, in order to support “situated health care responses” [28, p. 91].

To some extent, participatory approaches can also be applied in large-scale surveys in order to better reach certain populations. This was shown in the California Health Interview Survey in which participatory approaches informed many phases of study planning and implementation. This included several outreach activities informed by inputs “from many state and local public health agencies, health care organizations, the academic community, and advocacy groups through a series of public meetings, [and] key informant interviews […]” [44, p. 2].

In summary, the cited studies clearly demonstrate that collaborations between researchers, local groups, and public health institutions on various levels offer several methods for a joint definition of the relevant population which takes into account various levels of disease causation and public health intervention.

### Reconsidering context

“Throughout the history of public health, depending on the theory of disease causation prevalent at the time, different aspects of individuals and their environments have been considered important as potential causes of disease” [12, p. 216]. Accordingly, the notion of context has changed over time: Until the 1990s, it was criticized that epidemiology did not include group or macro-level variables in study design [12, 62, 80]. As a response, multilevel modeling has become a common approach, but the integration of such models in an overarching design remains challenging, given that each level interacts with different contexts [81]. Ecological models are also being applied, accounting for various contexts by applying interdisciplinary approaches to address issues of complexity [18]. Also, social epidemiology and cultural epidemiology are exploring historically grown contexts by examining locally constructed meanings of health and illness [78] and by studying socially developed practices and their impact on health-related outcomes [45].

In participatory research, context plays a central role. Local contexts are emphasized, requiring that the study design be adapted to the place, as well as to the interests and needs of the co-researchers from the population “under study” both in the interest of achieving health equity. Such a contextualization of research has a strong impact on modeling. This was demonstrated in a participatory evaluation model to develop health statistics for the state of New Mexico (USA). Contextualizing took place by integrating “private and public agencies, community groups, schools, higher education, and tribal entities […]” through “community-based decision-making and improved service coordination” [46, p. 199f]. Another example of contextualization is the cross-sectional survey on knowledge, behavior and attitudes regarding HIV, viral hepatitis, and sexually-transmitted infections among immigrants from Sub-Saharan Africa in Germany. The Robert Koch Institute (the national public health institute in Germany) is applying a participatory research design to collect data in different German cities, together with community representatives of the researched population. Several strengths of the communities are being recognized as being both important to the context and important for the research outcome, such as peer support in the form of advice from community members on certain behavior and attitudes [49].

It should be noted, however, that contextualization through a participatory process can cause conflicts. Conflicting goals among the diverse stakeholders—communities, policy makers, activists, and researchers—are not uncommon. And conflicts can arise regarding the translation and implementation of knowledge into local structures. Both aspects are reported in the evaluation study on health statistics in New Mexico (cited above) [46]. This study demonstrates that participatory research is challenged by asymmetric goals, needs, and power relations among the stakeholders. Interestingly, another study in the same context reported that the commitment among the stakeholders applying a participatory approach clearly outweighed these conflicts. By acknowledging the value of intermediate outcomes the collaboration can be sustained [82].

Epidemiologists can profit from such experiences, being inspired by how to contextualize their goals, questions, and methods. This is especially important in research with communities who have rejected the usual epidemiologic instruments as being inappropriate, as was reported in a study with First Nations in Canada [47]. Another way which the principle of contextualization is being applied is to prioritize research topics through citizen deliberation [48]. By using participatory research methods, epidemiologists can explicitly take into account contextual factors in order to make their research and the produced knowledge more applicable and relevant to practice.

### Synthesizing heterogeneous data

In health research various sorts of qualitative data (e.g. narratives) and quantitative data (e.g. statistical data) are being retrieved to answer research question concerning individuals, groups, localities, regions or even world-wide systems (e.g. global health). The applied methods for synthesizing data differ, depending on the underlying research paradigm such as positivism (e.g. experimental design), constructivism (e.g. interpretative design) or critical theory [25, 83].

Epidemiology is characterized by a positivist stance. This means that data collection and analysis is focused on factors which can be measured in a strict sense (quantification). Epidemiologic research has developed standardized statistical methods of increasing sophistication, basing the explanatory power of a study on the mathematically tested strength of relationships between the variables in various forms of modelling. The challenge lies in finding the “right” instruments to maximize predictive performance [84]. In the interest of feasibility (and parsimony), exposure-outcome-relations are modeled using a restricted set of variables, which may include social and other contextual factors. Even sophisticated methods of quantitative research are limited, however, as they have difficulty capturing problems related to disease and health for which quantitative data is not available or for which there are severe limitations to measurement.

By applying participatory approaches, epidemiologists can adopt a “realistic” stance, and, thus, a more grounded approach to the generation of data. Actually, this is not new in epidemiologic research practice. In the 1960s and 1970s, holistic approaches were developed to incorporate social and cultural factors in community-based models of disease [11]. In the last two decades, ecological modeling has become increasingly popular as a way to take into account complex circumstances in theory building and research design [5, 10, 18, 25].

Participatory epidemiology, as presented in this paper, also depends on such models. In general, participatory research utilizes heterogeneous data from different sources addressing different levels (e.g. individual, communities, networks). In the research process, either qualitative or quantitative methods can be applied, or both. The involvement of various stakeholders in the research process necessitates additional criteria for internal and external validity. These include, amongst others, intersubjective validity (the extent to which the research is viewed as being credible and meaningful by the stakeholders) or catalytic validity (the extent to which the research is useful in terms of presenting new possibilities for public health action) [24]. These forms of validity are aimed at assuring the relevance of the research for all those involved, so that the findings can be used directly to address public health issues.

For example, the method *neighborhood mapping* or *community mapping* is a well-established participatory approach which is also common in epidemiology. Such methods are suitable to describe the “social geography” of certain populations, for example to generate data on reported network size or ethnographic data, as demonstrated in a study with gay and bisexual communities in Vancouver, Canada [50]. Neighborhood mapping was also applied in an ecological study on urban infant mortality to inform program development in Baltimore, Maryland (USA). Based on a participatory community evaluation, a conceptual model was developed which comprised physical (e.g. built environment), social (e.g. organizations, norms, behavioral systems) and individual (e.g. characteristic of the mothers) factors. This model informed data collection (geocoding and map generation) to create community-based indicators for certain risks regarding infant mortality [51]. Even though environmental variables are already frequently included in epidemiologic modelling [52], participatory forms of mapping enable epidemiologists to obtain a more fine-grained data [53].

In summary, participatory research provides a framework that allows new ways for retrieving and synthesizing heterogeneous data through a collaborative process.

### Managing the research process

In common epidemiologic approaches, the steps in a research process are planned and driven solely by academic experts following a predefined research protocol to ensure valid findings. In participatory approaches, research is planned and conducted by academic researchers together with public health practitioners and community partners [64]. This is made possible by applying a set of participatory practices for the co-production of knowledge, which often have the dual goal of producing epidemiologic evidence while contributing to concrete public health interventions [26, 54]. The academic researcher is often in the role of facilitating the research process, or s/he can serve as a consultant or team partner in a research process managed by practitioners and/or community groups. The close relationship between the generation of evidence and the design of interventions to affect health outcomes has several implications for the temporal order and the dynamics of the research process. The development of an appropriate protocol, including the choice and application of methods, is an integral part of the collaborative research process. And the knowledge gained is intended to have an immediate relevance for advancing practice on specific health issues. Accordingly, participatory approaches are characterized by a cyclical, iterative process of development, implementation, adaptation, and interpretation by academics, practitioners, and representatives of the researched population [64].

For example, the data quality for both scientific purposes and for public health practice can be enhanced by introducing a flexible design that allows integrating various perspectives on a certain health topic. This was demonstrated in a study on the relationship between exposures to toxic chemicals and thyroid disorders applying a community-oriented design: “Such a[n] [approach] would foster communication and prevention measures within communities often left out of the dissemination of information about risks identified in studies conducted with residents of these communities” [71, p. 863]. Another example is the US-based National Children’s Study [26]. Participatory principles informed several steps of the research process including planning (e.g. focus groups complemented by literature reviews), study development (e.g. joint development of community engagement strategies), recruitment and data acquisition (e.g. a peer research strategy), and the dissemination of findings (e.g. community-level publications and educational programs). Participatory management of this sort can also be applied in smaller research projects, as seen in a study on variances in mortality rates caused by cervical cancer. A “culturally sensitive cervical health survey” [55, p. 67] was developed in several steps, applying participatory principles to better explain variances between certain groups of Native American and Caucasian American in an area in the Great Plains (USA). These studies demonstrate that participatory research in epidemiology can be strongly connected with democratic principles that enable and sustain dialog and joint planning.

In summary, the adaptive nature of participatory research, which allows utilizing data for various purposes, makes participatory approaches valuable to use in epidemiology.

### Disseminating findings

It is well understood that the translation of epidemiologic knowledge into “appropriate policy, programs, and interventions [is] inherently tricky, and often politically controversial” [56, p. 375]. A recently published review of epidemiologic textbooks concludes that even such publications do “not readily extend to methods suitable for assessing public health problems and priorities” [21, p. 1]. Even social epidemiologists can fail to give relevant recommendations for policy makers or other stakeholders in public health practice [85]. There is a longstanding and controversial discussion on the responsibility of epidemiologists to disseminate findings in a way that goes beyond providing information and advice [56, 57]. Thus, questioning and discussing the mandate and the future role of epidemiology in public health continues to be relevant [9, 61].

One crucial aspect of dissemination is the relevance of the findings. Relevant are findings which connect the realms of academic research, policy, and public health practice [86]. Participatory research approaches strive explicitly for a high level of relevancy among the stakeholders by applying dialogical methods for the co-production and dissemination of knowledge [58]. Participatory research also offers several ways for epidemiologists to disseminate their findings beyond the scientific community. For example, by using focus groups to find appropriate criteria to communicate genetic research information, as shown in a study with native people in Alaska (USA) [2] or by developing culturally appropriate recommendations for public health interventions, as shown in a study with First Nations in Canada [47].

In summary, participatory research is an approach that can help to disseminate epidemiologic findings through communicative venues with a reach beyond the scientific community. These venues can be organized locally by stakeholders, setting an example for participatory research management in other contexts. In addition, since participatory research involves the population “under study” in research, the dissemination of findings can be achieved earlier and can also reach the co-researching populations more directly [87].

Table 2 provides a summary of the foregoing results. With regard to the seven aspects of the research process, we compare the most common approaches to epidemiologic research and participatory approaches to epidemiology.

**Table 2:** Seven aspects of participatory research in epidemiology

Aspects	Common epidemiologic practice	Participatory epidemiologic practice
Defining the research goal	– Identification of molecular, cell-level, individual, group-level, and environmental risk-factors – Identification of social and other determinants of health – Specific focus preferred	– Identification of individual, group-level, and environmental health promoting factors – Aims to change social and other determinants of health – Strives for comprehensiveness
Defining the research question	– Driven by academic agenda, political imperatives, or unforeseeable events – Research object defined by professional system – Questions developed by scholarly persons	– Driven by group-level or local needs, political agenda, or unforeseeable events – Research subject defined by professional and lay system – Questions developed by scholars, practitioners, and/or lay persons
Defining the population	– Statistically relevant attributes applied – Individual level criteria preferred – Social criteria applied (group level and higher levels) – Macrosocial criteria applied	– Socially and politically relevant attributes applied – Individual criteria may be considered – Relies on social criteria (group and local level preferred) – Macrosocial criteria maybe applied
Reconsidering context	– Research on supranational, national, regional, and local level – Environmental, cultural, or social contexts may inform modeling – Multilevel modeling preferred	– Local level preferred, regional or state level may be considered – Environmental, cultural, or social contexts explicitly inform modeling – Ecological modeling preferred
Synthesizing heterogeneous data	– Leading paradigm quantitative (complemented by qualitative methods) – Data collection and analysis oriented towards measurable factors – Specific validity criteria (granted by standardized methods)	– Leading paradigm qualitative (complemented by quantitative methods) – Data collection and analysis oriented towards local and/or systemic change – Specific validity criteria (granted by equitable research principles)
Managing the research process	– Research is planned and driven by scholarly persons – Predefined research protocols applied – Stringent sequencing of steps ensures quality	– Research is planned and driven collaboratively – Recursively adapted research protocols applied – Adaptive sequencing of steps ensures quality
Disseminating findings	– Various formats (scientific publications, reports, advisory services) – Implementation usually delegated (depending on mandate)	– Various formats (educational programs, community based initiatives, scientific publications, reports) – Implementation in practice (alongside research process)

## Discussion: advancing participatory epidemiology

As with other forms of participatory health research, participatory epidemiology is an emergent science. As such, several issues need to be addressed if the approach is to be applied on a wider scale [6, 70, 73]. These issues include the following:

### Making use of existing data

An initial step in several participatory health projects is finding ways to use existing data for the purpose of defining the health issue and/or for the purpose of measuring the impact of interventions. This includes both surveillance data and more general epidemiologic research on health risks. Surveillance data is often not available at the level of community interventions [88], requiring additional data gathering or an extrapolation of findings from a higher level of aggregation to the locality under study. The application of other research is also limited by the frequent lack of contextualization, as described above. However, general findings identifying risks in certain groups or places can be an important starting point for specifying the research goals and questions in a participatory epidemiologic study. Making use of existing data requires creating ongoing collaborations between public centers for epidemiology, academic institutions, and those conducting research.

A good example for such a strategy is the online data collection and monitoring system for New Mexico’s Community Health Councils (USA), which was developed collectively by applying a participatory evaluation design. The result is a system which has relevance for both public health professionals and the wider community [82]. Participatory planning and other dialogical methods ensured the development of locally suitable and relevant indicators. Epidemiologists can engage in such projects to develop locally relevant indicators. Furthermore, such monitoring systems can contribute to the “regionalization” of data, which is still a challenging task in epidemiology [89, 90].

### Capacity building on the local level

The limited applicability of existing data to local contexts requires capacity building at the local level for collecting and analyzing epidemiologic data. This, in turn, requires a close collaboration between public centers for epidemiology, academic institutions, and those conducting research at the local level. Capacity building relates to different organizational levels [91, 92] and may involve actors pursuing different objectives [93]. However, academic researchers, public authorities, public health practitioners, and representatives of local communities can work together to develop “collaborative capacity” [94]. In participatory research, such collaborations are starting points for the identification of locally relevant health topics and the development of collaborative practices for data collection, analysis, and planning [95]. To take action, these collaborations also need to develop “agency capacity”, in order to address, for example, certain risks in vulnerable populations [96]. Participatory approaches to research can frame such endeavors by applying reciprocal research practices [97].

For epidemiologists, engaging in such collaborations can bridge the gap “that exists between those who use computational data and those who use [often locally based] cultural and linguistic models to generate their explanations” [67, p. 1135].

### Expanding the repertoire of methods

As described above, the type of data typically gathered in epidemiologic research can lack crucial information which stakeholders need to address public health issues. Particularly information related to social and political determinants of health are often missing, and such information is often best captured in mixed methods or qualitative studies, producing narratives of how health problems arise and how they can be alleviated [98, 99].

In participatory research various methods can be integrated [24]. This can result in a generation of data with a higher level relevance, for example, regarding sensitive and stigmatized health issues [55, 100] or the reconstruction of individual and group-level legacies influencing the health status of certain populations [78]. This methodical enhancement is possible because participatory research promotes the systematic reflection on underlying power relations in the research process through dialog, recursive methods of understanding, and joint planning. Community-validated measures are, however, not necessarily valid and reliable in a broader sense, at least not in the eyes of academic researchers, because the community partners are focused on promoting change in their specific context [101]. Academic researchers are thus challenged to be flexible in choosing, sequencing, and adapting research methods in a way which may not meet their usual standards.

One example for an expanded epidemiologic approach is the development of a monitoring system of fine particles and black carbon in Roxbury, MA (USA), where various media, stakeholder meetings, and community-level educational programs have been used to develop an appropriate and acceptable methodology [40].

### Applying multiple perspectives in data synthesis

There have been tremendous gains in the theory and practice of data synthesis over the last 20 years, with the pooling of large data sets and various forms of meta-analysis allowing for more powerful studies examining the causes of disease and health [102, 103].

Most relevant for participatory forms of research are approaches such as mixed methods reviews [104] and realist reviews [105] which are based on various forms of triangulation [103]. These approaches can enhance data synthesis in different ways. Firstly, by taking into account the heterogeneity of available data, such as program data, surveillance data, data from smaller studies, and narratives from the research populations (*data triangulation*). Secondly, by integrating and utilizing the perspectives of academic researchers and the co-researching practitioners and community members (*investigator triangulation*). Thirdly, by applying different theories on study design, e.g. theories that describe local health issues (*theory triangulation*). Fourthly, if needed, by combining and sequencing different methods for data synthesis (*method triangulation*) [106, 107]. Such approaches may also include the aforementioned participatory forms of validation.

Epidemiologists can profit from such approaches when local knowledge (e.g. expert opinion and the experiences of communities) is needed to conduct research to support public health interventions [103]. There is still much work to be done on developing these approaches. But their use is important if epidemiologists and local researches are to gain the most from the wide range of data sources, methods, and theories available for their work.

### Making explicit the theoretical foundation

There is a longstanding debate regarding which theoretical basis is appropriate for epidemiology, with several claiming that the lack of theory is one of the central problems epidemiology faces [5, 10, 74]. Approaches like the ecosocial theory of disease distribution [8, 23] are addressing explicitly and comprehensively the various sociopolitical dimensions of health. As such, they provide an ideal theoretical basis for participatory epidemiology with its concern for the social and political causes of health and disease and how to address them. These causes include, for example: social policy; social structures; social determinants as factors measurable at the individual level; psychosocial exposures; history; and biological pathways of embodiment. The ecosocial theory not only grounds participatory epidemiologic studies in a larger body of knowledge, but it also honors and supports researching disease and illness in a larger context.

In this sense, an integrated social–ecological systems lens was applied in a study on dengue in Dhaka (Bangladesh) to define infectious disease drivers [18]. By applying interdisciplinary theories and systems thinking, researchers were able to explain causes and determinants of dengue on different systems levels. Additionally, recommendations on the household, municipality, and regional levels were provided.

### Adding quality criteria

Epidemiologic evidence rests upon quality criteria such as randomization, controlling for confounders, and replicable study protocols as part of a large battery of standardized research procedures [108]. Given that participatory epidemiology differs in method and theory from several other epidemiologic approaches, it needs to set quality criteria which will necessarily differ in some respects from the usual epidemiologic standards. At the same time, participatory research provides quality criteria which “control for” factors often disregarded in common study designs. These criteria, as formulated by the International Collaboration for Participatory Health Research [24], are primarily concerned with ensuring equitable participation within the research process and describing the primary characteristics of such a process.

For example, the appraisal of evidence in epidemiology is generally determined by specific norms for sampling, data collection and analysis, and also by a relatively distant positioning of the researchers as related to the people they are studying [109]. In participatory approaches, the shared decision-making in the research process often means making changes to typical data collection routines, with the advantage of generating evidence that is meaningful for academic researchers, practitioners, and community members [73, 85, 86].

### Limitations of the review

There are some limitations to consider regarding the results and the methods of this study. The main limitation is the extent of theoretical and methodical variation in the included studies underpinning our framework. Since there is no consistent body of literature regarding participatory approaches in epidemiology, the provided framework cannot claim to be comprehensive, overlooking certain domains of the research process. We therefore can only provide preliminary definitions that need further clarification. The search process within the scoping review also had limitations: First, there was an imbalance between scientific publications and grey literature (e.g. reports). Since many epidemiologic investigations are conducted outside the scientific community, for example by local or regional public health agencies, more effort should be made to explore this field. The grey literature could only be included in a limited way in our study, due to access issues. Second, we only included publications in English and German and therefore systematically excluded publications in other relevant languages.

## Conclusion

Since public health “is about disease prevention and health promotion, lifestyle practices, cultures, the environment, social forces, historical traditions, and science in all its theoretical, methodological, and technological splendor”, epidemiologists can collaborate with many partners to “acquire scientific knowledge that matters to public health and to apply the knowledge gained in public health practice” [110, p. 1804]. As outlined in this paper, participatory epidemiology embraces this longstanding thread [59, 74, 111] and places emphasis on the mutual benefits of participatory approaches for academic researchers, public health practitioners, and the co-researching representatives of the populations being studied.

We have reviewed a large range of literature regarding stakeholder participation and other recent developments in the field of epidemiology, seeking to answer to questions of how participatory approaches are applied in epidemiology and what distinguishes them from non-participatory research, with the purpose of describing the implications for epidemiologic research practice. We found that participatory research partnerships in epidemiology can generate new, more comprehensive and more widely meaningful knowledge which can be applied more easily to make positive changes in people’s health. The framework developed in this paper is meant to encourage epidemiological researchers and their partners in applying participatory principles to their work as a way to bridge the gap between description and action.

Currently, we are applying some aspects of our framework in PartKommPlus–German Research Consortium for Healthy Communities [112]. In PartKommPlus, participatory research is being applied to study the factors influencing the implementation and maintenance of health promotion strategies in German municipalities. In this context, the Robert Koch Institute (RKI) has established collaborations in order to explore participatory approaches to health reporting and epidemiology at both the municipal and the national levels.

### Key messages

- Participatory epidemiology is a conceptual framework to enrich methodology. It offers ways to better contextualize epidemiologic research and provides more detailed definitions of the population under study. It is useful for working with heterogeneous data. It facilitates collaborative practices and offers innovative ways to disseminate findings.
- Participatory epidemiology fosters dialogue and partnership in research by allowing various frames of reference. This results in evidence which is useful for both academic researchers and public health practitioners.

## Abbreviations

HIV: human immunodeficiency virus
NIDDM: non-insulin-dependent diabetes mellitus
STI: sexual transmitted infections
